# Holographic Focused Ultrasound Hyperthermia System for Uniform Simultaneous Thermal Exposure of Multiple Tumor Spheroids

**DOI:** 10.3390/cancers15092540

**Published:** 2023-04-28

**Authors:** Diana Andrés, Ian Rivens, Petros Mouratidis, Noé Jiménez, Francisco Camarena, Gail ter Haar

**Affiliations:** 1 Instituto de Instrumentación para Imagen Molecular (I3M), CSIC—Universitat Politècnica de València, Camino de Vera S/N, 46011 Valencia, Spain; diaanbau@upv.es (D.A.); nojigon@upv.es (N.J.); fracafe@fis.upv.es (F.C.); 2 Institute for Cancer Research (ICR), London SM2 5NG, UK; ian.rivens@icr.ac.uk (I.R.); petros.mouratidis@icr.ac.uk (P.M.)

**Keywords:** acoustic holograms, acoustic lenses, ultrasound hyperthermia, tumor spheroids, thermal dose

## Abstract

**Simple Summary:**

Acoustic holograms were investigated as a method of delivering simultaneously controlled ultrasound hyperthermia to multiple tumor spheroids. Findings show that holographic lenses designed to deliver a uniform thermal dose rather than a uniform acoustic field can tune the natural focus of a transducer, allowing the exposure to ultrasound-mediated hyperthermia of several tumor spheroids simultaneously, and improving experimental throughput for future studies. Preliminary in-vitro findings suggest that hyperthermia delivered by ultrasound and polymerase chain reaction heating have different outcomes, with ultrasound being more lethal for the same thermal dose.

**Abstract:**

Hyperthermia is currently used to treat cancer due to its ability to radio- and chemo-sensitize and to stimulate the immune response. While ultrasound is non-ionizing and can induce hyperthermia deep within the body non-invasively, achieving uniform and volumetric hyperthermia is challenging. This work presents a novel focused ultrasound hyperthermia system based on 3D-printed acoustic holograms combined with a high-intensity focused ultrasound (HIFU) transducer to produce a uniform iso-thermal dose in multiple targets. The system is designed with the aim of treating several 3D cell aggregates contained in an International Electrotechnical Commission (IEC) tissue-mimicking phantom with multiple wells, each holding a single tumor spheroid, with real-time temperature and thermal dose monitoring. System performance was validated using acoustic and thermal methods, ultimately yielding thermal doses in three wells that differed by less than 4%. The system was tested in vitro for delivery of thermal doses of 0–120 cumulative equivalent minutes at 43 °C (CEM_43_) to spheroids of U87-MG glioma cells. The effects of ultrasound-induced heating on the growth of these spheroids were compared with heating using a polymerase chain reaction (PCR) thermocycler. Results showed that exposing U87-MG spheroids to an ultrasound-induced thermal dose of 120 CEM_43_ shrank them by 15% and decreased their growth and metabolic activity more than seen in those exposed to a thermocycler-induced heating. This low-cost approach of modifying a HIFU transducer to deliver ultrasound hyperthermia opens new avenues for accurately controlling thermal dose delivery to complex therapeutic targets using tailored acoustic holograms. Spheroid data show that thermal and non-thermal mechanisms are implicated in the response of cancer cells to non-ablative ultrasound heating.

## 1. Introduction

Hyperthermia, commonly defined as an increase in tissue temperature to between 39 and 45 °C, is a well-established form of cancer treatment with proven efficacy as a radio- or chemo-sensitizer [1]. When applied to the whole body, it has been observed that there is an enhanced immune response [2]. Various clinical techniques for delivering hyperthermia exist. These include electromagnetic, ultrasonic, conductive (via hysteresis of magnetic nanoparticles inside the body) heating, and hyperthermic perfusion [3]. Among these, ultrasound is the only non-invasive method capable of inducing highly localized hyperthermia inside the body without using endogenous contrast. By using focused ultrasound, the mechanical energy of an acoustic wavefront can be concentrated on the focal volume of the system, and tissue is heated locally, mainly through the activation of thermoviscous processes [4]. 3D cell aggregates known as spheroids mimic the in vivo response of tumors to treatments better than 2D monolayers [5]. Cancer cell response to hyperthermia delivered with good temperature accuracy using a polymerase chain reaction (PCR) thermal cycler has been studied in single-cell suspensions and 3D tumor models, showing significant differences between the two models [6,7,8]. However, such studies are potentially limited because they lack the non-thermal mechanisms by which ultrasound may affect cell growth. Experiments with ultrasound on tumor spheroids in vitro have been performed to study drug delivery [9,10] and, more recently, to study spheroid response to heating using different ultrasound parameters in a non-absorbing fluid medium [11].

Ultrasound-induced hyperthermia is commonly delivered using a focused ultrasound (FUS) transducer, which concentrates the mechanical energy into a focal spot. Therefore, in practice, achieving adequate uniform heating throughout targets larger than the focus requires multiple, repeated sonications in different positions [12,13] and/or sophisticated and expensive phased-array technology to steer the focus rapidly [14,15]. 

In recent years, novel techniques based on 3D-printed acoustic lenses or holograms used to shape and control the acoustic field have emerged. It has been demonstrated that holographic lenses can compensate for the phase aberrations produced by refracting layers, such as skull bones, producing aberration-corrected focal spots [16] or therapeutic acoustic images of arbitrary shape [17]. Specific biomedical applications of holographically-designed lenses include cell patterning [18], cavitation pattern generation [19], and local blood-brain barrier opening for targeted drug delivery in the brain [20]. Recently, acoustic holograms have been used to generate arbitrary thermal patterns [21]. However, using this approach, the temperature rise is not uniform throughout the whole target.

The aim of this present work was to create a novel ultrasound hyperthermia system based on acoustic holograms capable of delivering a uniform thermal iso-effective dose (TID) over complex targets and to use it initially to study how ultrasound hyperthermia affects tumor spheroids in vitro. Using this approach, several spheroids were treated simultaneously in order to provide repeatable exposure conditions over several experimental samples. A holder in which spheroids could be heated to a target TID with ultrasound while the temperature is being monitored in real-time was developed. The novel system, based on an ultrasound-absorbing (tissue mimicking) gel phantom with three miniature wells with ultra-fine wire thermocouples below each, was initially designed to characterize the hyperthermia-lens system thermally and subsequently to expose tumor spheroids to predefined, well-controlled TIDs. Two holographic lenses which split the transducer’s geometric focus into several foci were compared. The first lens was designed to provide acoustically similar foci that could be targeted on each spheroid containing well of the phantom. The second hologram was tailored to produce similar heating rates at each spheroid. The acoustic and thermal performances of the lenses were measured and compared with modeling predictions. The ability to deliver a uniform TID to multiple U87-MG cell spheroids was investigated. Their growth response and viability were compared to that of thermocycler-heated spheroids.

While this system has only been tested here on spheroids, a future aim of this work is to provide a hyperthermia system capable of providing uniform thermal doses over the entire volume of a pre-clinical in vivo tumor. However, an in vitro method capable of simultaneously heating a number of 3D tumor mimics (spheroids) placed in a phantom that represents the heating seen in soft tissues in vivo (using the IEC ultrasound tissue mimic) will allow more clinically relevant in vitro experiments to be undertaken, thus reducing the number of animals needed to establish appropriate treatment regimens. 

## 2. Materials and Methods

### 2.1. Holographic Ultrasound System Design

The ultrasound hyperthermia system consisted of a focused ultrasound transducer coupled to a holographically-designed, 3D-printed acoustic lens. The lenses were created by time-reversal methods using pseudo-spectral time-domain simulations [22]. Essentially, the method consisted of placing virtual ultrasound sources at any position within the desired target region where a focal peak was required (i.e., at the location of each well). Each of these sources is set with a tuned amplitude and phase, as explained below. The field was backpropagated to the holographic surface, located at the lens position in front of the transducer. Using a complex conjugated (time-reversed) version of the captured field, a physical lens was generated that encoded the phase information of the field in its morphology. The lenses were constructed using a spherical coordinate system; see Supplementary Materials in [21]. Finally, a forward simulation using the holographic lens geometry was used to determine the resulting acoustic field. The process is equivalent to that described in Ref. [17], but in this case, a final thermal simulation [21] was used to calculate the resulting thermal field and TIDs over the target. In addition, this work characterized the inhomogeneous transducer vibration along its surface and included this information in the time-reversal simulations for the lens design and evaluation (for more detail, see Appendix A).

**Figure 1:**
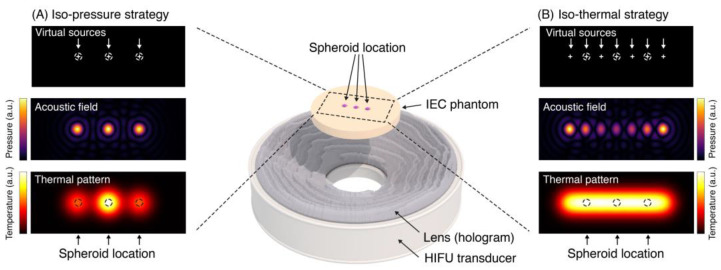
The two acoustic hologram design strategies. (**A**) Iso-pressure strategy, where the hologram is designed by setting a virtual source at each target location, resulting in a uniform acoustic pressure field at each target. (**B**) Iso-thermal strategy, where the lens is designed by locating a set of virtual sources of tuned amplitude and phase, covering the target locations, resulting in an uneven acoustic field but uniform thermal pattern.

Two design strategies were investigated, each of which aimed to produce an acoustic focus in individual spheroid-containing wells of the holder described below. Figure 1 summarizes both strategies. The first, acoustic (iso-pressure) strategy, aimed to achieve equal acoustic peak pressures at each of the spheroid locations (i.e., at each focus). However, due to heat diffusion, this very simple strategy may not yield a uniform thermal dose delivery to each spheroid. Therefore, a second, more complex approach to producing a uniform thermal distribution was investigated, the iso-thermal strategy. In order to produce a more uniform TID at each focal peak (spheroid) position, lower amplitude acoustic pressures were used more centrally, where the rate of cooling is lower, and higher amplitudes were used more laterally, where cooling was highest. For the iso-thermal approach, a first guess at a distribution of focal peak pressures was used, the acoustic field was modeled, and, from this, thermal simulation was performed until the medium arrived at equilibrium (a steady state temperature). This process was repeated iteratively, lowering the pressure in the areas where the temperature was still too high so as to approach a solution in which the thermal dose at each focal peak was the same. 

For the iso-pressure design strategy, as shown in Figure 1A, a 3-focus hologram with 2 mm focal separation was produced in order to obtain a similar acoustic field in each of the three spheroid wells. Three virtual sources were placed at a distance of *z* = 40 mm from the transducer surface, at the center on the *x*-axis (up, down), and at the well locations—2, 0, and 2 mm on the *y*-axis. During the iso-pressure design simulations, all virtual sources within the target region(s) emitted sinusoidal signals with the same amplitude and phase since the aim was to produce three foci with similar acoustic pressure.

For the iso-thermal strategy, as shown in Figure 1B, a 7-focus hologram with a separation of 1 mm between maxima was designed. Seven virtual sources were placed 40 mm (z) from the transducer surface at the center of the *x*-axis and at locations −3, −2, −1, 0, 1, 2, and 3 mm on the *y*-axis. Each of these emitted continuous sinusoidal signals with different amplitudes and opposed phases two-by-two. In this case, the whole backward and forward modeling process was repeated iteratively, thus tuning the virtual-source amplitudes to obtain a uniform thermal field by setting lower amplitudes for the central virtual sources accounting for thermal conduction from the lateral foci. Due to the long temporal duration of each iteration (each acoustic simulation lasted ~600 min, and each thermal simulation lasted ~300 min on an Intel^®^ Xeon^®^ CPU e5-2608 v2 workstation (Santa Clara, CA, USA), no optimization algorithm was applied, and source weights were tuned manually. Progressively reducing the amplitude of the central virtual source resulted in a uniform TID in the targets due to the compensation between the heating rate and heat transport mechanisms in different areas.

### 2.2. Holographic Lens Design

For all holograms, the interference of the acoustic waves produced by multiple virtual sources results in a complex acoustic wavefront. Using simulations, the resulting field, $H\left(\theta_{0},\phi_{0}\right)$ on a curved surface parallel to the transducer surface and located at a distance r=d, where $\theta_{0}$ and $\phi_{0}$ are the polar and azimuthal angles, respectively, is recorded. At this holographic surface, the acoustic lens should spatially modulate the source wavefront to match the complex-conjugated version of the recorded wavefront at the central frequency. 

Lenses are designed in spherical coordinates to match the semi-spherical shape of focused transducers. The holographic lens is divided into pixels, each having the shape of a small column of variable height protruding perpendicularly from the transducer surface. Each pixel is considered to be a longitudinal elastic resonator of height $h=h\left(\theta_{0},\phi_{0}\right)$. The complex transmission coefficient measured at a distance d perpendicular to the source surface is given by [17]

(1) $$T\left(\theta_{0},\phi_{0}\right)=\frac{2Z_{n}e^{-ik_{0}\left(d-h\right)}}{2Z_{n}\cos\left(k_{L}h\right)+i\left(Z_{n}^{2}+1\right)\sin\left(k_{L}h\right)}\;,$$

where $Z_{n}=Z_{L}/Z_{0}$ is the normalized acoustic impedance, $Z_{0}=\rho_{0}c_{0}$ and $Z_{L}=\rho_{L}c_{L}$ are the acoustic impedances of water and lens material, respectively, $\rho_{0,}$ is the water density, $\rho_{L}$ is the lens material density, $c_{0}$ is the sound speed of water, $c_{L}$ is the sound speed of the lens material, $k_{0}=\omega /c_{0}$ is the wavenumber in water and $k_{L}=\omega /c_{L}$ the wavenumber in the lens material at the central angular frequency ω.

**Figure 2:**
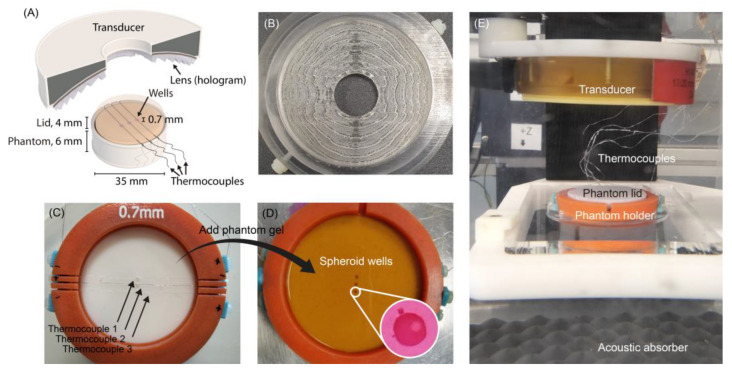
Holographically designed lens, high intensity focused ultrasound transducer, and absorbing gel spheroid holder used in hyperthermia experiments. (**A**) Schematic of the spheroid exposure system with its dimensions. (**B**) 3D-printed lens with the holder by which it is mounted on top of the transducer. (**C**) The mold was used to form the absorbing gel phantom with the three thermocouples located 0.5 mm above the protrusion used to form each well. (**D**) The mold was filled with IEC tissue-mimicking gel, with 3 miniature wells into which a spheroid could be placed (inset). A gel lid closed the phantom (**E**) Experimental setup to expose the spheroids to ultrasound hyperthermia.

The height of each lens pixel is obtained by interpolating the value of h that makes the measured complex-conjugated phase at the holographic surface equal to the phase value of the transmission coefficient, i.e., finding $h\left(\theta_{0},\phi_{0}\right)$ that gives $\arg\left(T\right)=\arg\left(H^{*}\right)$. The height function is obtained by numerical interpolation of Equation (1) [17]. Once the lens has been designed, it can be manufactured and attached to the transducer, as shown in Figure 2A. Stereolithography technology was used to 3D print the lenses using VeroClear resin (Objet30 printer (Stratasys, Israel)). An image of a lens used in this study is shown in Figure 2B.

### 2.3. Acoustic Field Validation

The acoustic field produced by the holographic lenses was measured using a hydrophone in a water tank filled with degassed (<3 mg/L dissolved oxygen) deionized water at room temperature (21 °C). The hydrophone (HNA-0400, ONDA Corp, Sunnyvale, CA, USA) was mounted on an automated 3D positioning system (UMS, Precision Acoustics, Dorset, UK). The 1.66 MHz source was a H148-MR piezo-ceramic transducer (Sonic Concepts Inc., Bothell, WA, USA) with a 63.2-mm radius of curvature, 64-mm outer diameter, and 23-mm inner hole diameter, mounted in a fixed position and driven with sinusoidal pulsed-burst signals (40 cycles, PRF = 200 Hz, 40 mVpp) from a signal generator (33250A, Agilent, Santa Clara, CA, USA), after 55 dB amplification (A300, E&I, Queens, NY, USA). Signals were recorded using a digital oscilloscope (64Xi, LeCroy, Chestnut Ridge, NY, USA) using a time window of 20 µs after reaching a steady state to avoid the ramp-up period. A total of 20 waveforms per measurement point were averaged. 

The uniformity of source vibration across the transducer surface, in the absence of the lens, was measured and used as input for the lens design and numerical evaluation. A 2D measurement in a plane parallel to the transducer face and 15 mm closer to the transducer than the focal peak was performed. The scan, designed to cover the active beam, was carried out from −12 mm to 12 mm in both x and y directions, with a spatial resolution of 0.3 mm and a signal generator setting of 50.2 mVpp. The measured field was back-projected to the transducer surface using the Rayleigh-Sommerfeld integration, as described by Sapozhnikov et al. [23]. 

To evaluate the acoustic field generated by the holographic lenses, software-controlled 2D scans were made through the central focal peak in a plane parallel to the transducer face, using a grid of 81 × 21 points and with a spatial resolution of 0.1 mm.

### 2.4. Thermal Exposure System

In order to evaluate the thermal profile produced with the holographic lenses and to subject (500 to 800 μm diameter) spheroids to a precisely controlled iso-thermal dose, a new exposure system was designed. The system needed to absorb ultrasound in a similar way to tissue, to heat spheroids, and be instrumented so that temperature and TID could be accurately monitored.

In order that several spheroids could be exposed simultaneously to the same TID, one spheroid was held in each of 3 separate miniature wells (0.7 mm deep and 1 mm diameter) spaced 2 mm apart, as shown in Figure 2C,D. Thermocouples (detailed below) were manually placed in the mold, aligned with the middle of each well but 0.5 mm below its bottom so they would be embedded in the gel. These provided an estimate of the temperature achieved in each well; see Figure 2A. Although 3 wells were chosen for this holder, the technique should also be applicable to a larger number.

Once the thermocouples were in place, the molds were filled with the acoustically absorbing IEC tissue mimic [24]. Phenol red (3%) was added during gel manufacture to improve the ability to visualize the spheroids in the well and to aid their removal after exposure. The dye had a negligible impact on gel acoustic properties as measured with an in-house system [25]. Spheroid holder bases were 4 mm thick and separate gel lids were made that were also 4 mm thick, so that the bottom of the spheroid containing well lay 5 mm beneath the top surface of the holder.

Thermal measurements were made with the spheroid holder in a fixed location, immersed in degassed water at a constant 38 °C, obtained using a recirculation circuit consisting of a water chiller (HC-100A, Hailea, Guangdong, China) and a degasser (2.5 × 8 Liqui-Cel Membrane Contactor, 3M, Saint Paul, MN, USA), connected to a vacuum line (27 in. Hg) plus a water heater (GD 100, Grant Scientific, Cambridgeshire, UK) placed in the exposure tank.

During exposures, temperature was recorded using the three embedded 75-μm diameter T-type thermocouples (COCO-003, Omega, Manchester, UK) connected to a high-density thermocouple module (NI-9213, National Instruments, Austin, TX, USA) used in high-resolution mode to obtain 1 measurement per second. The transducer was mounted on a 3D positioning system with a 60-micron movement resolution. The temperature resolution was ±0.1 °C. No attempt was made to compensate for potential thermocouple measurement artifacts, as viscous heating should be <25% of the measured values [26]. An image of the experimental setup is shown in Figure 2E.

Firstly, the transducer’s natural geometric acoustic focus was localized on the central thermocouple, using a grid of one-second sonications followed by nine seconds of cooling time, during which the transducer was moved to the next position. The three cartesian axes were interrogated separately using spatial steps down to 0.25 mm. The same protocol was followed to locate the other two thermocouples relative to the central one. 

Secondly, the lens was mounted on the transducer, and the axial distance was altered to reflect the expected difference in focal position (10 mm). The central thermocouple was re-localized as described. Four to five-minute exposures with a continuous signal from −1 mm to 1 mm with a spatial step of 0.25 mm along the *y*-axis were then performed, aiming to achieve an equilibrium state in temperature and to evaluate the TID received by the spheroids in each well. 

The thermometry system captured the temperature on the thermocouples as a function of time. TID was calculated as [27]

(2) $$TID=\overset{n}{\underset{i=1}{\sum }}t_{i}\cdot R^{43-T_{i}},$$

where TID is the thermal dose in units of cumulative equivalent minutes at 43 °C (CEM_43_), $t_{i}$ is the *i*-th time interval, $T_{i}$ is the average temperature during time interval $t_{i}$ and R is related to the temperature dependence of the rate of cell death, defined as *R* (T < 43 °C) = 1/4, *R* (T > 43 °C) = 1/2.

The in-house software used to measure and record temperature also displayed accumulated TID, thus enabling exposures to be halted once a pre-determined dose had been accumulated. Experiments in the absence of spheroids showed that a predictable and repeatable TID accumulated once exposures were halted. This was used to manually achieve TID delivery to spheroids. Spheroid exposures ended once one of the three thermocouples reached a TID of 120 CEM_43_ for the 3-focus lens and 60 CEM_43_ TID for the 7-focus lens.

### 2.5. Acoustic and Thermal Simulations

To evaluate the performance of the holographic lenses, acoustic and thermal numerical simulations were performed using k-Wave software [22]. All simulations used a 492 × 492 × 492-point grid, with a Perfect Matching Layer (PML) of 10 grid points on each side and with a homogeneous spatial resolution of *λ*/6, where *λ* is the wavelength in water (*λ* = 0.91 mm). The transducer was modeled as a spherical cap with a central hole, each point emitting a continuous sinusoidal signal of amplitude and phase given by the measured ceramic vibration. The medium was considered to be homogeneous, with water properties of temperature = 38 °C, density $\rho_{0}$ = 1000 kg/m^3^, sound speed $c_{0}$ = 1520 m/s, and attenuation coefficient $\alpha_{0}$ = 0.0023 dB/(cm-MHz^2^). Water temperature was set to match that used in the experiments, and water density, sound speed and attenuation were calculated according to references [28,29,30], respectively. The lens was 3D printed using VeroClear resin properties measured in the laboratory with pulse-echo, finite-amplitude insertion-loss techniques: $\rho_{L}$ = 1181 kg/m^3^ and $c_{L}$ = 2525 m/s, and with acoustic absorption set to $\alpha_{L}$ = 1.65 dB/(cm-MHz^γ^), with γ = 1.1 the attenuation power-law frequency exponent, as reported in [31]. A total simulation time of 50 µs was used, with a 20 ns time step, and the maximum peak pressure amplitude in the last three cycles in a steady state was recorded at all grid points to give a steady state pressure map for comparison with the experiment.

Thermal simulations were based on a pseudo-spectral time-domain numerical solution of Penne’s bio-heat equation [32]. For the in vitro system, phantom dimensions and characteristics were used, and it was assumed to be surrounded by water. Its acoustic properties were set to $\rho_{p}$ = 1050 kg/m^3^, $c_{p}$ = 1540 m/s, values reported by the IEC standard (IEC 60601-2-37:2007). Phantom-specific heat capacity and thermal conductivity were set to $C_{p}$ = 3550 J/(kg·K) and $k_{p}$ = 0.52 W/(m·K), respectively, while for the water, these parameters were $C_{0}$ = 4185 J/(kg·K) and $k_{0}$ = 0.62 W/(m·K). Cooling due to perfusion was not relevant. After studying the convergence of the simulation, heating times up to 10 min were simulated, with time steps of 0.5 s. The initial temperature was set to 38 °C in all media. At each time step, the water temperature was set to this initial value to replicate experimental conditions in which the water was maintained at a constant temperature. Pressure at each point was set to that measured with the hydrophone in water to match experimental conditions. The volume rate of heat deposition in the medium was defined as $Q=\alpha_{p}p^{2}/\rho_{p}c_{p}$. The relationship between the temperature (T) difference from baseline (T_0_), pressure (*p*), and the volume rate of heat deposition (Q) was found to be $T=2\cdot 10^{7}Q/p^{2}+T_{0}$, and the attenuation value of the phantom that best matched the simulation with the experiment was $\alpha_{p}$ = 0.67 dB/(cm-MHz^γ^). This was used in the thermal simulations for both lenses.

### 2.6. Tumor Spheroid Exposure

U87-MG cells (ATCC, London, UK) were maintained as a sub-confluent monolayer at 37 °C in 75 cm^2^ flasks in a humidified atmosphere containing 5% CO_2_. They were propagated using Dulbecco’s modified Eagle medium (DMEM) supplemented with 10% hybridoma cell culture grade fetal bovine serum (SIGMA, Poole, UK), 2 mM L-glutamine, 50 U/mL penicillin, 50 mg/mL streptomycin B, 0.25 lg/mL amphotericin B and sub-cultured using Accutase (SIGMA, Poole, UK). Screening for mycoplasma contamination was carried out on a monthly basis. Spheroids were created by plating 20,000 U87-MG cells in the wells of ultra-low attachment (ULA) U-shaped well plates (96-well 7007, Corning, NY, USA). Cells were allowed to aggregate for 48 h to form spheroids. 

For ultrasound exposure, spheroids were transferred from the U-shaped well plates to the empty phantom’s wells using wide-bore pipette tips (Alpha Laboratories, Eastleigh, UK). Work was carried out aseptically where possible, and antibiotics, as described above, were used to minimize the risk of contamination. To maintain spheroids in a fixed location inside the phantom wells, they were sealed using 4 μL of matrigel. The matrigel (and spheroids) were allowed to set for 10 min, and then the lid was placed on the phantom. The spheroids were then exposed to ultrasound hyperthermia. Two sets of three tumor spheroids (six in total) were exposed to TIDs of 7.5, 30, 60, or 120 CEM_43_. Eight spheroids were placed in the wells and placed in the exposure tank, but not exposed to ultrasound (sham exposures). Spheroid sizes ranged between 0.5 and 0.8 mm at the time of exposure. TID was controlled by monitoring temperature (and TID) in all 3 wells in real-time. As the central well approached the intended TID, the exposure was halted manually to take into account the probable dose accumulation during cooling, determined during measurements made in the absence of spheroids. The cooling phase contributed < 2.4 ± 0.2% of the total dose. At the end of the cooling phase, the phantom was dismantled, and spheroids were pipetted back into the ULA plates. These plates were then incubated in a humidified incubator at 37 °C, 5% CO_2_. 

For thermocycler heating, a calibrated PCR thermal cycler (C100 Touch Thermal Cycler, Bio-rad, Hercules, CA, USA) utilizing a Peltier thermoelectric system to transfer thermal energy was used to expose U87-MG spheroids to TIDs of 0, 30, 60 and 120 CEM_43_ using temperatures of 37, 44, 45 and 46 °C, respectively, for 15 min as described in our previous publications [5,6,7]. A TID of 7.5 CEM_43_ was achieved by heating spheroids to 43 °C for 7.5 min. Spheroids were then incubated in ULA plates in the same way as ultrasound-heated spheroids. 

### 2.7. Size and Adenosine Triphosphate Measurements

For size measurements, the mean diameter of each spheroid was measured with a high-throughput digital microscope (Celigo Image Cytometer, Nexcelom, Lawrence, MA, USA) using high accuracy filters, automated high brightness focus, only 1 spheroid per well, and pixel intensity of 0–120. Spheroid pictures were also collected using the Celigo. The Cell Titer Glo 3D assay (Promega, Madison, WI, USA) was used as described in the supplier’s handbook to provide a relative measure of the intracellular ATP (adenosine triphosphate) present in the spheroids and thus indicate their viability via this measure of metabolic activity. Briefly, spheroids in 100 μL media were transferred to white 96-well flat bottom plates and incubated with 25 μL of Cell Titer Glo for 30 min at room temperature with gentle agitation. A signal from each bubble-free well was immediately detected using a luminescent microplate reader (FLUOstar Optima, BMG Labtech, Ortenberg, Germany). Data were then processed in Excel.

### 2.8. Statistical Analysis

Microscopy size results are presented as mean ± standard deviation (SD) of *n* = 3 spheroids for thermocycler heating experiments, and for ultrasound-heating experiments, *n* = 8 for sham exposed spheroids, *n* = 5 for 7.5, 30, and 60 CEM_43_ exposed spheroids and *n* = 3 for 120 CEM_43_ exposed spheroids. Statistical significance was calculated using a 2-way unpaired Student’s t-test assuming unequal variance. *p* values < 0.05 were considered statistically significant. 

## 3. Results

### 3.1. Lens for Iso-Pressure Focal Peaks (3-Focus)

**Figure 3:**
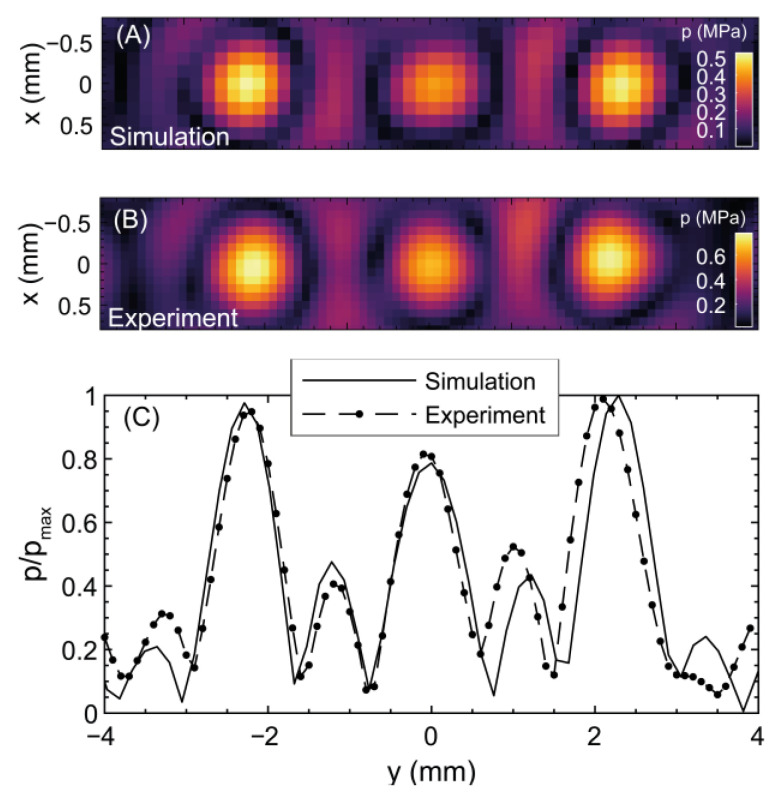
Acoustic pressure field for the three-focus hologram in the x-y plane at z = 40 mm: (**A**) simulation (**B**) experimental hydrophone measurement. (**C**) normalized hydrophone measured *y*-axis plot through the pressure maxima.

Figure 3A shows a transverse 2D plot through the central acoustic focal peak of the 3-focus (acoustic iso-pressure strategy) lens modeled in water. The comparable measured acoustic field at 40 mVpp driving voltage is shown in Figure 3B. Peak positive pressures measured at each focus (left to right in the figure) were 0.84 ± 0.05, 0.7 ± 0.05, and 0.8 ± 0.02 MPa and matched well with the simulation. Errors were obtained as the standard deviation between the maximum peak pressure over the 20-cycle signals at the focus. A normalized linear plot through the central focal peak is shown in Figure 3C. The three foci have FWHM values of 0.85 ± 0.05, 0.89 ± 0.05, and 0.91 ± 0.05 mm and were deviated +0.1, −0.1, and −0.2 mm relative to their simulated locations on the *y*-axis. The two lateral foci had the highest pressure, with the central focus being 15% lower amplitude. For thermal exposures where 85 mVpp driving voltage was used, the peak positive pressures at each focus were 1.34 ± 0.08, 1.12 ± 0.08, and 1.28 ± 0.03 MPa.

As described earlier, each thermocouple in the phantom holder was localized using the transducer’s geometric focus. Their positions, relative to the central one (on the *z*-axis), were (0, −2, 0) ± 0.25 mm and (0, 2, 0) ± 0.25 mm. Using the 3-focus holographic lens and continuous exposures at 85 mVpp, sonications were performed moving the transducer from −1 mm to 1 mm around the central location, with spatial steps of 0.25 mm and exposure times of around 5 min, until the central thermocouple reached a TID of approximately 120 CEM_43_. 

**Figure 4:**
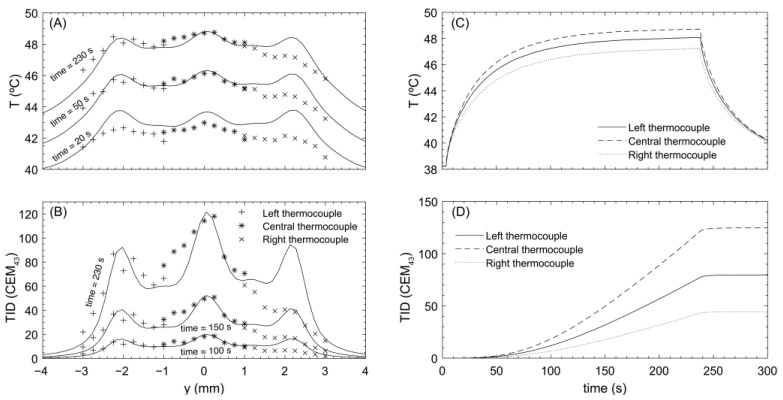
Lens for iso-pressure focal peaks. (**A**) Temperature profile at different time points simulated (solid line) and measured experimentally with each thermocouple. (**B**) TID profile at different time points simulated (solid line) and measured experimentally with each thermocouple. (**C**) Experimental temperature profile over time at the central position measured by each thermocouple. (**D**) Experimental TID profile over time at the central position measured by each thermocouple.

Thermal profiles recorded at each localized thermocouple are shown in Figure 4. Maximum focal peak temperatures were 48.0 ± 0.1, 48.7 ± 0.1, and 47.2 ± 0.1 °C, from left to right, respectively. These values correspond to TIDs of 72.8 ± 6, 114.3 ± 9, and 41 ± 3 CEM_43_ after 5 min of continuous heating. The mean TID in each well was calculated as the mean TID in measurements made between −0.5 and 0.5 mm around the central location for each thermocouple, as this is the uncertainty in the spheroid location, and the error in this value is obtained as the standard deviation of these measurements. With this calculation, mean TIDs at each well were 73 ± 13, 103 ± 14, and 38 ± 6 CEM_43_, from left to right. Comparing the mean TID in the central well with the others gives a relative error of 29.1% for the one located at its left and 63.1% at its right, while the difference between the left and the right wells was 47.9%. 

### 3.2. Lens for Iso-Thermal Dose (7-Focus)

**Figure 5:**
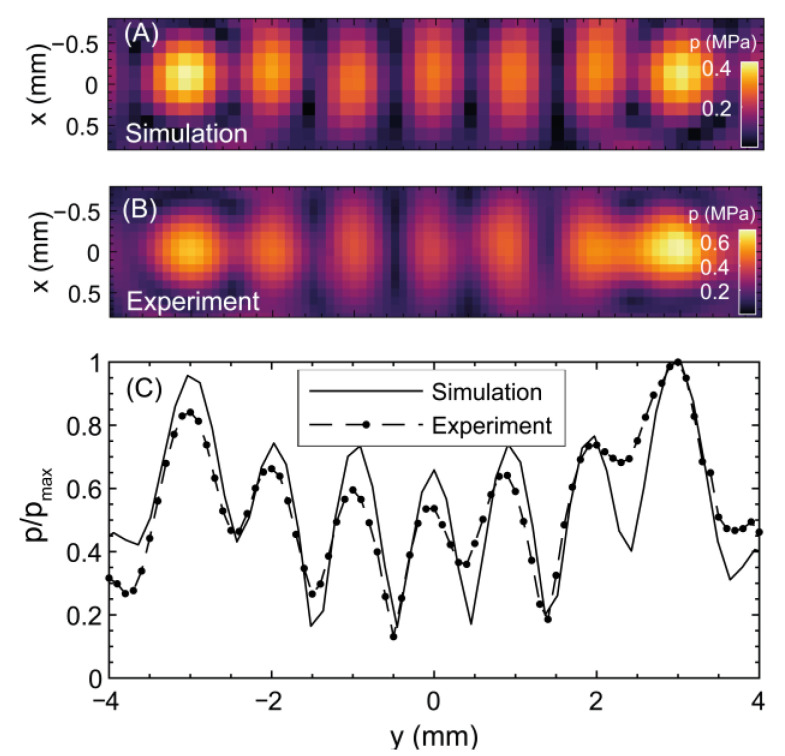
Acoustic pressure field for the seven-focus hologram in the x-y plane at z = 40 mm: (**A**) simulation (**B**) experimental hydrophone measurements. (**C**) normalized hydrophone measured *y*-axis plot over the pressure maxima.

Figure 5A shows a simulated transverse 2D plot through the focal peak of the central focus of the 7-focus lens and the measured acoustic field at 40 mVpp driving voltage (see Figure 5B. Peak positive pressures at each focus were (left to right): 0.65 ± 0.06, 0.50 ± 0.05, 0.49 ± 0.05, 0.41 ± 0.05, 0.54 ± 0.03, 0.55 ± 0.04, and 0.68 ± 0.03 MPa. These matched well with the simulation. A normalized linear cut through the acoustic maximum is shown in Figure 5C. Seven focal spots separated by 1.0 ± 0.1 mm appeared in the same locations as in the simulation. There is a difference in maximum acoustic pressures of up to 40% between the seven foci. For thermal exposures, where 85 mVpp driving voltage was used, the peak-positive pressures at each focus were 1.04 ± 0.09, 0.80 ± 0.08, 0.78 ± 0.08, 0.66 ± 0.08, 0.86 ± 0.05, 0.88 ± 0.06, and 1.09 ± 0.05 MPa, from left to right.

The same protocol as with the 3-focus lens was followed. With the 7-focus lens-focused transducer system, continuous exposures at a drive of 85 mVpp, designed to achieve 60 CEM_43_ TID at the central thermocouple, were used. These sonications were performed as described above., using exposure times of ~7.5 min. 

**Figure 6:**
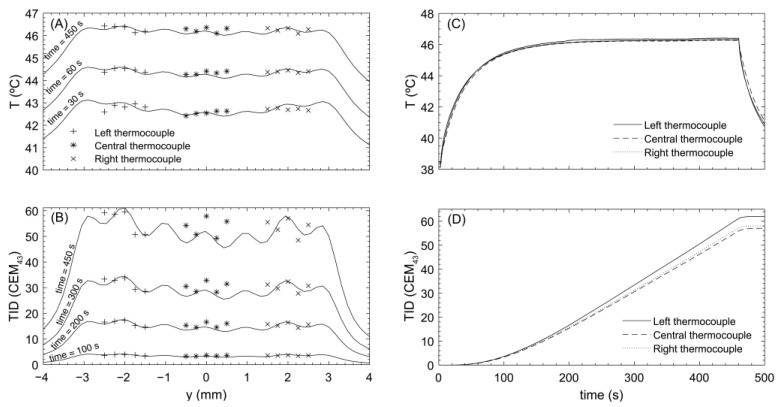
Lens for iso-thermal dose distribution. (**A**) Temperature profile at different time points simulated (solid line) and measured experimentally for each thermocouple. (**B**) TID profile at different time points simulated (solid line) and measured experimentally for each thermocouple. (**C**) Experimental temperature profile over time at the central position measured by each thermocouple. (**D**) Experimental TID profile over time at the central position measured by each thermocouple.

The temperature-time profile recorded at each thermocouple is shown in Figure 6. The maximum steady-state temperatures registered by each thermocouple were 46.4 ± 0.1, 46.4 ± 0.1, and 46.3 ± 0.1 °C, from left to right. These values correspond to measured TIDs of 60 ± 5, 58 ± 5, and 57 ± 5 CEM_43_. Mean TIDs (calculated as described for the 3-focus lens) for each well were 56 ± 5, 54 ± 4, and 54 ± 3 CEM_43_, from left to right. Comparison of the mean TID in the central well with the others gives a relative difference of 3.7% for the one located to its left and 0.03% to its right.

### 3.3. Spheroid Study

**Figure 7:**
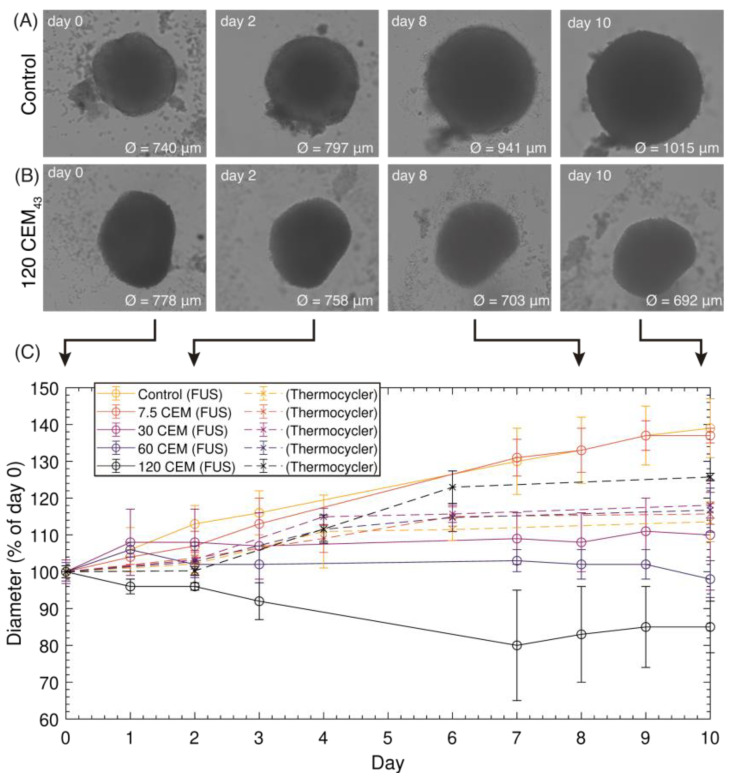
Images of a control spheroid (**A**) and a 120 CEM_43_ treated spheroid (**B**) on days 0 (day of treatment), 2, 8, and 10. (**C**) Growth curves for the control spheroids and those treated with ultrasound or thermocycler heating, as a % of their diameter on day 0. Results are presented as mean ± SD.

U87-MG tumor spheroids were exposed to ultrasound heating using TIDs of 7.5, 30, 60, and 120 CEM_43_ (six for each condition). Due to early inexperience with the use of pipettes for removing spheroids from the wells, five were retrieved for each condition, except for those exposed to 120 CEM_43_, for which three were retrieved. Eight spheroids were sham-exposed. Figure 7A,B show the evolution over 10 days of a sham-exposed spheroid (growth) and an ultrasound-induced 120 CEM_43_ exposed spheroid (shrinkage), respectively. Figure 7C shows growth curves for ultrasound- and thermocycler-heated spheroids over a 10-day period. Sham- and 7.5 CEM_43_ ultrasound-exposed spheroids continued to grow, increasing their size by approximately 40 ± 4% in 10 days. Shrinkage of spheroids by approximately 15 ± 6% was seen when they were ultrasound-treated to a TID of 120 CEM_43_, whereas no shrinkage or growth was seen at 30 or 60 CEM_43_. No shrinkage was seen in the thermocycler-heated spheroids exposed to TIDs of 0, 7.5, 30, 60, or 120 CEM_43_.

**Figure 8:**
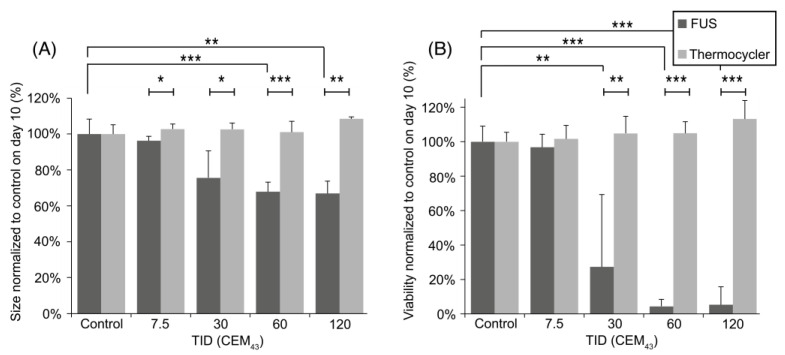
(**A**) Size and (**B**) viability study on day 10 after treatment for FUS and thermocycler-treated spheroids. Results are presented as mean ± SD, and statistical significance is denoted with an asterisk: (*) *p* ≤ 0.05, (**), *p* ≤ 0.01, (***), *p* ≤ 0.001.

Ten days after treatment, U87-MG spheroids exposed to ultrasound showed a greater reduction in growth than those heated using a thermocycler (Figure 8A). For example, the size of spheroids exposed to ultrasound at TID of 120 CEM_43_ was 33 ± 5% smaller than that of sham ultrasound-treated spheroids 10 days after treatment. When the same TID was delivered to the spheroids using thermocycler heating, their size did not present a significant reduction compared to sham-exposed spheroids at this time point (Figure 8A). Differences in size between HIFU and thermocycler-heated spheroids are statistically significant for all TIDs.

The metabolic activity of spheroids, indicative of their viability, was also assessed 10 days after heat treatment. Figure 8B shows a statistically significant reduction in spheroid viability when exposed to ultrasound-heating with TIDs of 30, 60, and 120 CEM_43_ (with relative viability of 27 ± 42%, 4 ± 2%, and 5 ± 10%, respectively, compared to sham-exposed spheroids). Non-statistically significant differences in viability were seen when spheroids were exposed to US heating TIDs of 7.5 CEM_43_ (97 ± 4%) compared to sham-exposed spheroids. Thermocycler-heated spheroids showed no significant changes in their viability when exposed to TIDs in the range of 0–120 CEM_43_. Significant differences (*p* < 0.05) are seen when comparing the viability of HIFU-exposed spheroids with thermocycler-heating for TIDs of 30, 60, and 120 CEM_43_ (Figure 8B).

## 4. Discussion

Two different lens design strategies for achieving simultaneous iso-thermal ultrasound hyperthermia treatments of tumor spheroids in vitro have been compared. An acoustic (iso-pressure) strategy with a 3-focus holographic lens and an iso-thermal strategy with a 7-focus one. The 3-focus lens produced acoustic pressure peaks at the desired location, with similar peak pressures (differences less than 15%), which led to peak temperatures of 47.9 ± 0.8 °C in the phantom material. Since differences in temperature greater than ±0.3 °C yield differences in TID of ± 20% [6,7] it turned out not to be possible to design an appropriate lens with three foci based solely on acoustic modelling. In fact, readings over the three thermocouples showed that, after five minutes of exposure, each had received significantly different TIDs: 72.8, 114.3, and 40.5 CEM_43_. Whilst this allowed simultaneous exposure of 3 spheroids, they did not receive similar TIDs. 

Using the 7-focus lens, the acoustic profile was tuned to be more inhomogeneous than the iso-pressure case, with lower pressure in the central peaks than in the lateral ones (40% difference between the central peak and the external ones). However, this tuned acoustic field produced a more uniform TID in the targets. The thermal field from the 7-focus lens achieved a temperature of 46.37 ± 0.17 °C over the three thermocouples, with a TID of 58.2 ± 1.3 CEM43, which represents a great improvement relative to the iso-pressure strategy where differences in TID received by the spheroids were of ± 37 CEM43. This was successful because the central region cooled more slowly due to thermal conduction from the lateral foci and therefore required a lower rate of energy deposition, as accounted for in the lens design, unlike the iso-pressure lens, where the same pressure was set for the three foci resulting in a higher steady-state temperature at the central focus than in the other two.

Exposure of the tumor spheroids and evaluation of the TID they were receiving in real-time, and ultimately stopping treatment when an intended dose was achieved, worked well with the 7-focus lens. The newly designed spheroid holder, which used an ultrasound absorbing (IEC) tissue-mimicking gel with three wells and a separate lid, which was tested here, generally worked well. This system allowed the location of the spheroids within the wells to be known to be better than +/−0.25 mm relative to the thermocouple locations. The phantom with the lid maintained the heat and prevented spheroids from exiting the well. Using this system, it was possible to expose three spheroids simultaneously to ultrasound heating with the accuracy of temperature, and thus TID, monitoring demonstrated here. It was shown that ultrasound heating of the spheroids using TIDs of 30 to 120 CEM_43_ resulted in reduced spheroid growth, associated with lower metabolic activity (viability), compared to either sham-exposed controls or thermocycler-heated spheroids. These results show that ultrasound heating improves tumor spheroid growth control compared to thermocycler heating and is suggestive of the contribution of non-thermal mechanisms to the anti-cancer effect of heat, such as cavitation, which locally enhances the heating rate in the exposed region [33] or acoustic radiation forces, which have been proven responsible for cell deformation [34]. The main technical challenge was that spheroid retrieval from the wells using a pipette was difficult, resulting in the loss of 25% of spheroids after treatment, even after changing the gel color from grey to pinkish/orange to improve the color contrast with the white spheroids.

This system also has other limitations. The height of the pixels of the 3D-printed holographic lens will not be completely precise but will depend on the tolerance of the printer. Additionally, driving the transducer in continuous mode results in lens heating, which can change its geometry and the polymer properties. On the other hand, regarding the exposure system, the phantom holder must be held rigidly in a fixed and reproducible position relative to the transducer-lens system and be removed and replaced when spheroids are changed. Another difficulty is the location of the fine-wire thermocouples just below and in the center of the well, which needs to be completed manually and gives an error on their exact location. The phantom gets degraded when pipetting spheroids in and out of the wells and when exposing it to ultrasound heating, so it needs to be changed quite often, ideally each time a new experiment is to be completed. Additionally, spheroids need to be removed from their sterile environment for being exposed to ultrasound, even though all manipulation and system mounting is completed in a sterile manner.

The expansion of the clinical applications of HIFU seen in recent years has resulted in an increase in the number of pre-clinical in vivo studies. This hologram-based system can contribute to a reduction in the use of research animals because it provides a reliable, inexpensive, and quick method of exposing appropriate tumor models to ultrasound heating, thus providing an opportunity for the investigation of their biological and immunological responses. It is also amenable to studies investigating hyperthermia enhancement of radiotherapy.

Iso-thermal dose delivery systems are also of future interest for treating tumors in vivo with a uniform TID over the whole tumor volume. To design a holographic system for such conditions, a number of challenges need to be addressed. For example, in-vivo models are complex biological systems of different shapes, sizes, and anatomical locations subjected to variable levels of blood perfusion. Although complicated, thermodynamic modeling is possible, as shown by elegant studies performed by Kaczmarek et al. [35], who have used a range of blood perfusion rates to predict lesion sizes in a model of ultrasound-induced hyperthermia in the presence of magnetic nanoparticles. Additionally, it is possible to modify the tissue-mimicking phantom used in this study to include an artificial blood vessel [36]. Hence, for future investigations, our approach can be extended to generate a uniform 3D thermal pattern using a 3D distribution of virtual acoustic sources taking into account the expected heat-sinking effect caused by blood perfusion. For designing the optimal acoustic hologram, fast optimization techniques to tune the amplitude and phase of these acoustic sources will be required.

## 5. Conclusions

Using a hologram designed to produce a 3-focus acoustic field with equal focal peak pressures, i.e., an iso-pressure design strategy, did not deliver simultaneous uniform TID to spheroids contained in 3 individual wells because of reduced cooling by thermal conduction from the central well. The 15% maximum difference in peak pressure foci resulted in TID differences of up to 63%.

In order to achieve a similar TID within each well, an iso-thermal strategy was used to design holograms that produced multiple focus acoustic fields with different pressures at each focal peak, resulting in a uniform TID. Using this approach, differences of 40% in focal peak pressure were found, but TID, with differences of less than 4%, could be delivered.

A case study was performed with U87-MG tumor spheroids to study their response to different TIDs with this last acoustic hologram. The biological response showed greater anti-cancer effects for ultrasound-mediated heating than for thermocycler heating, suggesting that our system provides a reasonably high throughput platform for investigating the biological responses of tumor spheroids in vitro to ultrasound hyperthermia.

These results show that holographic lenses designed to deliver iso-thermal doses coupled to single-element HIFU transducers can provide simultaneous uniform TIDs in complex targets or over relatively wide regions using a low-cost but robust system. Further work could be carried out to optimize thermal uniformity in different complex 2D or even 3D targets by, for example, applying optimization and machine learning algorithms [37] to accurately engineer the acoustic wavefront and produce uniform thermal patterns for ultrasound hyperthermia systems.

## Figures and Tables

**Figure A1:**
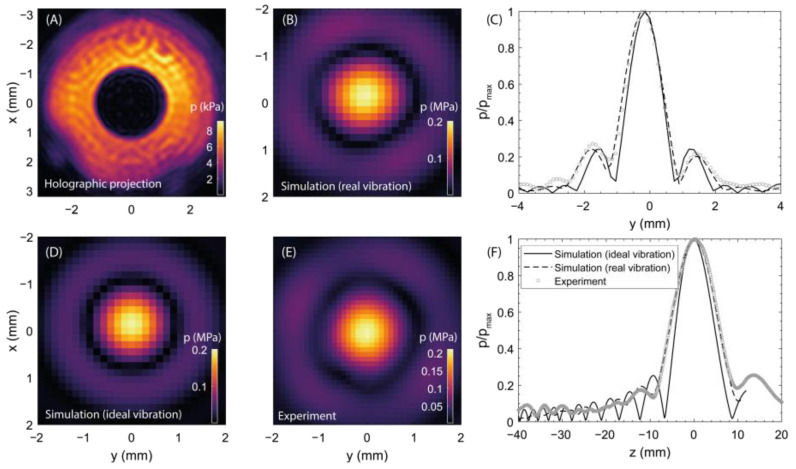
(**A**) Back projection of the measured acoustic pressure onto the transducer surface, showing the pressure distribution over the transducer surface. (**B**) Simulation of the focal acoustic pressure using the ceramic’s actual vibration. (**C**) x-cut through the focal peak (simulations and experiment). (**D**) Simulation of the focal acoustic pressure assuming homogeneous surface vibration. (**E**) Experimental measurement. (**F**) z-cut through the acoustic maximum (simulations and experiment).

**Figure A2:**
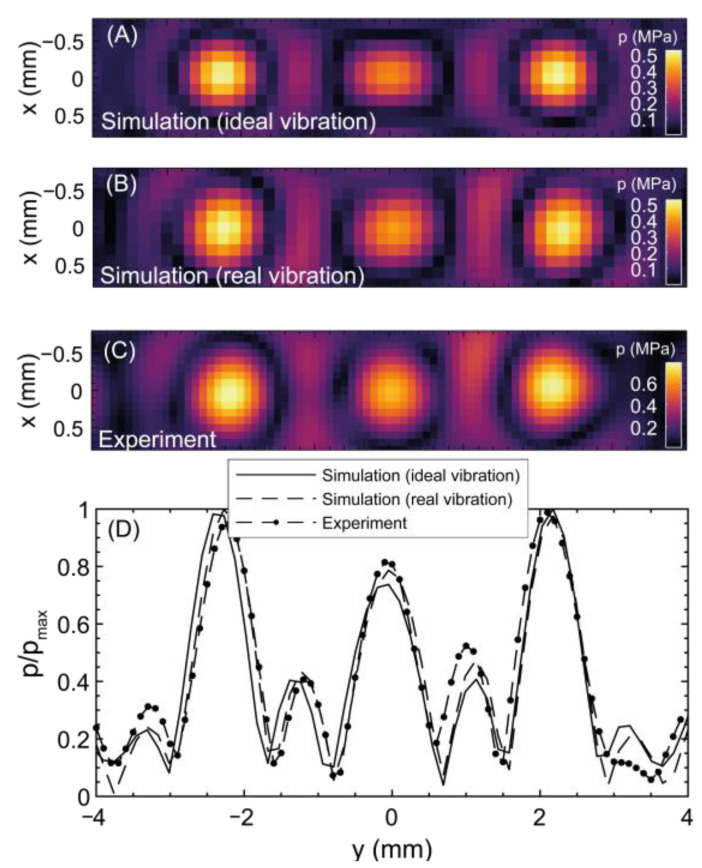
(**A**) Acoustic simulation of the XY plane at z = 40 mm (acoustic maximum) for the 3−focus hologram, assuming homogeneous transducer surface vibration. (**B**) Comparable acoustic simulation using the actual ceramic vibration. (**C**) Comparable experimental acoustic measurement. (**D**) Normalized linear plot over the hologram maximum peak pressure in the XY−plane.

**Figure A3:**
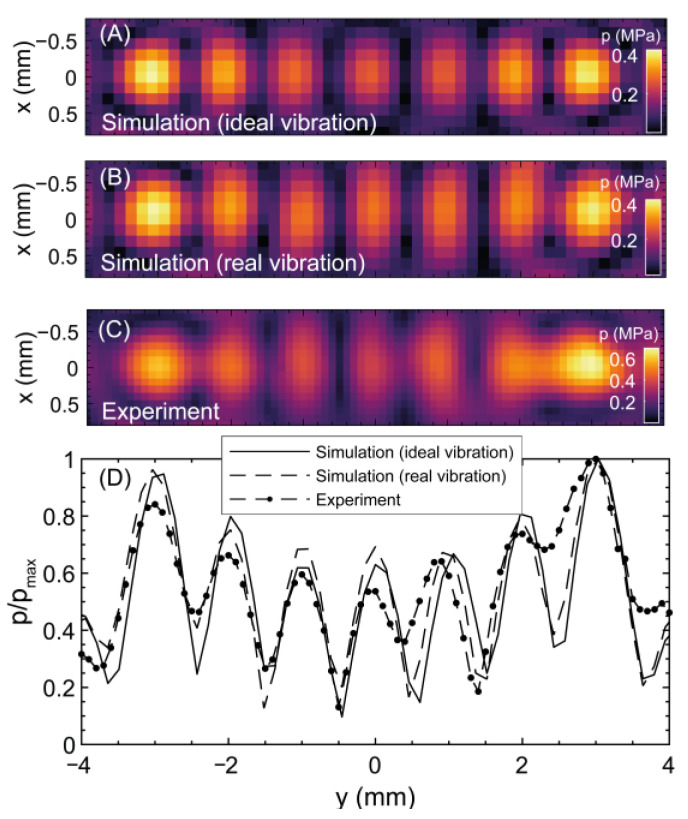
(**A**) Acoustic simulation of the XY plane at z = 40 mm (acoustic maximum) for the 7−focus hologram, assuming homogeneous vibration. (**B**) Comparable acoustic simulation using the real ceramic vibration (**C**) Comparable experimental acoustic measurement. (**D**) Normalized linear plot over the hologram maximum peak pressure in the XY−plane.

## Data Availability

The data that support the findings of this study are available from the first author, D.A., upon reasonable request.

## Appendix A

### Transducer Vibration Evaluation

In order to gain an understanding of how the transducer ceramic was vibrating in practice, needed for improved acoustic simulation of the transducer-lens system compared to an assumption of uniformity across the entire surface, holographic measurements of the acoustic pressure were made at the transducer surface. Measurements of the acoustic pressure between the focus and the transducer surface were made in a plane (XY) parallel to the transducer face, 15 mm from the focal peak. The scan was performed from −12 mm to 12 mm in both X and Y directions, with a resolution of 0.3 mm at a driving voltage of 50.2 mVpp. The measured field was projected back to the transducer surface using the Rayleigh-Sommerfeld integration as described by Sapozhnikov et al. [23]. This projection is shown in Figure A1. Using this data, a forward simulation was performed to evaluate the focus shape and amplitude. Results of this calculation were compared with those obtained for homogeneous vibration of the whole transducer, and with direct experimental measurements at the peak-pressure position. The maximum peak acoustic pressure measured with the hydrophone at the focus was 0.21 MPa, and the same value modelled using the real back-projected amplitude of the transducer was 0.2 MPa. Discrepancies between these values may be due to the uncertainty in the geometry of the ceramic, which was modelled to have the diameter and radius of curvature given by the manufacturer. FWHM values for the homogeneous vibration simulation, real vibration simulation and measured data, respectively, were 1.15 ± 0.1, 1.24 ± 0.1 and 1.3 ± 0.1 (x direction), 1.15 ± 0.1, 1.33 ± 0.1 and 1.24 ± 0.1 (y direction), and 9.1 ± 0.1, 11.6 ± 0.1 and 11.7 ± 0.1 (z-direction).

The results show that ceramic vibration affects the resulting acoustic hologram and so it is important that it is well characterized in order to achieve a better match between simulation and experiment.

In the case of the 3-focus hologram (Section 3.1), the maximum peak location is better predicted than when considering the transducer as a homogeneous vibrating source, while experimentally the three foci were found at −2.1, 0 and 2.2 mm, they were found at −2.4, 0 and 2.2 mm in the homogeneous vibration simulation, and at −2.2, 0 and 2.2 mm in the inhomogeneous vibration simulation. There was a difference of 3.4% in the central focus experimental peak pressure when compared to the inhomogeneous simulation and 9.5% for the homogeneous case (see Figure A2).

For the 7-focus hologram (Section 3.2), little difference in the focus location were appreciated. Each focus directly measured appeared at −3, −2, −1, 0, 0.9, 2 and 3 mm, while simulated with the real inhomogeneous ceramic vibration they appeared at −3, −2, −1, 0, 0.9, 2 and 3 mm and while simulated with homogeneous vibration they were at −2.9, −2, −1, 0, 1.1, 2 and 3 mm (see Figure A3). In view of these results, the inhomogeneous vibration of this ceramic is not very critical for slightly complex holograms, but the hologram performance changes as the complexity of the holographic lens increases and it is also dependent on the relative location of the lens to the transducer.
